# Association of Multiple Genetic Variants with the Extension and Severity of Coronary Artery Disease

**DOI:** 10.5935/abc.20170177

**Published:** 2018-01

**Authors:** Simone Cristina Pinto Matheus Fischer, Simone Pires Pinto, Lívia Campos do Amaral Silva Lins, Henrique Tria Bianco, Carlos Manoel de Castro Monteiro, Luiz Fernando Muniz Pinheiro, Francisco Antonio Helfenstein Fonseca, Maria Cristina de Oliveira Izar

**Affiliations:** Universidade Federal de São Paulo (UNIFESP), São Paulo, SP - Brazil

**Keywords:** Coronary Artery Disease / genetic, Polymorphism, Genetic, Metabolic Syndrome, Sedentary Lifestyle

## Abstract

**Background:**

Metabolic syndrome (MS) is a condition that, when associated with ischemic heart disease and cardiovascular events, can be influenced by genetic variants and determine more severe coronary atherosclerosis.

**Objectives:**

To examine the contribution of genetic polymorphisms to the extension and severity of coronary disease in subjects with MS and recent acute coronary syndrome (ACS).

**Methods:**

Patients (n = 116, 68% males) aged 56 (9) years, with criteria for MS, were prospectively enrolled to the study during the hospitalization period after an ACS. Clinical and laboratory parameters, high-sensitivity C-reactive protein, thiobarbituric acid reactive substances, adiponectin, endothelial function, and the Gensini score were assessed. Polymorphisms of paraoxonase-1 (PON-1), methylenotetrahydrofolate reductase (MTHFR), endothelial nitric oxide synthase (ENOS), angiotensin-converting enzyme (ACE), angiotensin II type 1 receptor (AT1R), apolipoprotein C3 (APOC3), lipoprotein lipase (LPL) were analysed by polymerase chain reaction (PCR) technique, followed by the identification of restriction fragment length polymorphisms (RFLP, and a genetic score was calculated. Parametric and non-parametric tests were used, as appropriate. Significance was set at p < 0.05.

**Results:**

Polymorphisms of PON-1, MTHFR and ENOS were not in the Hardy-Weinberg equilibrium. The DD genotype of LPL was associated with higher severity and greater extension of coronary lesions. Genetic score tended to be higher in patients with Gensini score < P50 (13.7 ± 1.5 vs. 13.0 ± 1.6, p = 0.066), with an inverse correlation between genetic and Gensini scores (R = -0.194, p = 0.078).

**Conclusions:**

The LPL polymorphism contributed to the severity of coronary disease in patients with MS and recent ACS. Combined polymorphisms were associated with the extension of coronary disease, and the lower the genetic score the more severe the disease.

## Introduction

Ischemic heart disease and stroke account for the majority of deaths in the world.^1^ With progressive urbanization, adoption of a sedentary lifestyle and better access to packaged food, increasing incidence of obesity and overweight has been observed in the population,^2^ accompanied by an increase in metabolic disorders and cardiovascular risks. The concept of metabolic syndrome (MS) was first described by Reaven, as a relationship between insulin resistance, arterial hypertension, lipid abnormalities and visceral obesity.^3,4^

MS has been associated with higher rates of fatal and non-fatal cardiovascular events.^5,6^ Its high prevalence in acute coronary syndrome (ACS) has also been associated with greater anatomical obstruction.^7^ Our group showed that patients with MS and ACS had lower insulin sensitivity, severe coronary artery disease (CAD) associated with high levels of C-reactive protein (CRP) and low IgG antibody titers to oxidized low-density lipoprotein,^8-10^ associated with greater extension of CAD.^11^

Some studies have explored the association of genetic variants with cardiovascular outcomes.^12-16^Large Mendelian randomization studies have allowed the understanding of the effects of some clinical parameters throughout life, such as LDL-c, HDL-c and triglycerides on cardiovascular risk, as well as the protective effect of polymorphisms associated with lower systolic blood pressure.^17-19^ Although hypothetical, the effects of polymorphisms have been associated with the extension and severity of CAD.^20-22^

Smaller studies, systematic review and meta-analyses have proposed genetic variants associated with CAD, as well as genetic scores to identify individuals at higher cardiovascular risk,^23-25^ since many genetic variants with only modest individual effect may have a larger impact when a greater number of genetic polymorphism is evaluated. Therefore, the use of genetic scores that evaluate the additional effect of each of these genetic variants may be relevant in the early identification of individuals at higher cardiovascular risk, who may benefit from differential attention.^23-25^

We chose polymorphisms related to lipid metabolism, lipoprotein oxidation, blood pressure changes and vasoreactivity to determine their possible association with coronary atherosclerosis severity in patients with MS and recent ACS. Studies on genetic polymorphisms in MS patients who developed ACS are poorly described in the literature, which reinforces the importance of the present study. We examined the contribution of combined and isolated genetic polymorphisms of potential importance in cardiovascular disease, by a genetic score, to the extension and severity of coronary obstructive disease in patients at very high cardiovascular risk.^26^

## Methods

### Subjects

A total of 116 consecutive patients of both sexes, mean age 56 ± 9 years, with 3 or more criteria for MS according to the NCEPIII^4^ were prospectively assessed during hospitalization for ACS (acute myocardial or unstable angina).

**Inclusion criteria:**

1. Men and women aged from 30 to 75 years;
2. Understanding and agreement in giving written consent.
3. Two or more ACS criteria including chest pain, increased enzyme levels and electrocardiographic changes;
4. Three or more MS criteria,^4^
5. LDL-c < 130 mg/dL, HDL-c < 40 mg/dL in the first 24 hours of hospitalization;

**Exclusion criteria:**

1. Use of hypolipidemic agents in the last 30 days;
2. Uncontrolled hypothyroidism (TSH > 8.0 *µ*U/mL);
3. Infections, inflammatory diseases, active liver or kidney diseases;
4. Pregnant women, patients selected for surgeries, including myocardial revascularization in the next six weeks or patients who underwent surgeries during hospitalization.

### Ethical aspects

The study protocol was approved by the Research Ethics Committee and the study was conducted according to the Helsinki Declaration. All patients signed the informed consent form before participating in any study procedure.

### Study design

This was a prospective study that included hospitalized patients after ACS. Genetic polymorphisms related to lipid and lipoprotein metabolism, oxidative stress, endothelial function and blood pressure were evaluated.

### Clinical evaluation

Clinical evaluation and blood collection for laboratory measurements were performed in the first three days of hospital discharge. Demographic data, risk factors, medical history, clinical examination, anthropometric data (weight, height, waist circumference), systolic and diastolic blood pressure were obtained.^27,28^

### Blood collection for laboratory tests

Blood was collected after 12 hours of fasting and laboratory tests were performed at the *Associação Fundo de Apoio à Psicobiologia* (AFIP - Psychobiology Support Fund Association). Genetic tests were carried out at the Molecular Biology Laboratory of the Division of Lipids, Atherosclerosis and Vascular Biology.

### Biochemical tests

Lipid profile (total cholesterol, LDL-c, HDL-c, triglycerides) was analyzed by automated method (Ópera, Bayer, Germany); glucose levels were determined by colorimetric reaction (ADVIA 1650 Chemistry System, USA), glycated hemoglobin (HbA1-c) was determined by high performance liquid chromatography (HPLC, Tosoh A1c 2,2 plus, USA). Mean insulin at -15min, -5min and 0min was assessed by direct chemiluminescence method (ADVIA Centaur, USA) with values expressed as µU/mL. Apolipoproteins A1, B and Lp(a) were analyzed by nephelometry (R100 analyzer, Behringer). Adiponectin was determined by ELISA (enzyme-linked immunosorbent assay), using the Humam Adiponectin/Acrp30 Immunoassay kit - Quantiquine, R&D Systems, and the BioTek ELx800 Absorbance Microplate Reader, with values expressed as ng/mL.

Albuminuria was assessed in 12-hour overnight urine samples, and measured by immunoturbidimetric method (ADVIA 1650, Chemistry System, USA), with results expressed as mg/L.

Plasma oxidative stress was analyzed by TBARS (Thiobarbituric acid reactive substances) test, as described by Ohkawa et al.,^29^ and measured using a spectrophotometer (Genesys 2, Spectronic); the results were expressed as nanomoles of malondialdehyde (MDA) per milliliter of plasma (hmoles/mL plasma).

### Vasoreactivity

Endothelium-dependent (FMD, flow-mediated dilation) and endothelium-independent (EIR, endothelium-independent relaxation) vasodilation tests were performed in the morning, after a 12-hour fast by an experient ultrasound technician according to the International Brachial Artery Reactivity Task Force guidelines.^30^ An ultrasound system (Sonos 5500; Hewlett-Packard-Phillips, Palo Alto, CA) was used, equipped with a vascular software for two-dimensional imaging, color and spectral Doppler, internal electrocardiogram (ECG) monitor and a linear array transducer (with a frequency of 7.5-12.0 MHz). Image acquisition, FMD and EIR measures with isosorbide dinitrate (5mg; sublingual) were performed. Percentage variations in vessel diameter were calculated for FMD and EIR determination, and expressed as percentage. Intra- and inter-observer variability were < 1% and 2%, respectively.

### Gensini score

Gensini score was used to classify CAD severity determined by cineangiography. Gensini score is calculated taking into account the magnitude of the lesions and the myocardium at risk, assigning different ratings to different levels of obstruction in the segments affected. A 25%, 50%, 75%, 90%, 99% and 100% obstruction were given, respectively, the scores 1, 2, 4, 8, 16 and 32. The method also assigned a different score depending on the lesion location - left coronary trunk (5 points), proximal anterior descending artery (2.5 points), middle third of the anterior descending artery (1.5 point), distal anterior descending artery (1 point), second diagonal artery (0.5 point), proximal and distal right coronary artery and posterior descending branch (1 point). Gensini score results from the sum of individual scores given to each lesion, multiplying the stenosis severity by the lesion location; results were expressed as arbitrary units (AU).^11,31^

### Genetic study

Polymorphisms of the genes paraoxonase-1 (*PON-1*), methylenotetrahydrofolate reductase (*MTHFR*), endothelial nitric oxide synthase (*ENOS*), angiotensin-converting enzyme (*ACE*), angiotensin II type 1 receptor (*AT1R*), apolipoprotein C3 (*APOC3*), lipoprotein lipase (*LPL*) were analyzed from total blood samples, collected with EDTA (ethylenediaminetetraacetic acid), using the polymerase chain reaction (PCR) technique, followed by the identification of restriction fragment length polymorphisms (RFLP), under conditions described in Table 1. Genetic score was calculated assuming the dominant model of the polymorphisms, which were considered as binary, dichotomous variables.

**Table 1:** Conditions for amplification and digestion of the studied polymorphisms

Polymorphism	Starters	Annealing temperature	Cycles	Restriction enzyme
*PON-1*	*Forward*: 5'-TATTGTTGCTGTGGGACCTGAG-3'	61°C	35	Alw I
Q192R	*Reverse*: 5'-CACGCTAAACCCAAATACATCTC-3'			
*MTHFR*	*Forward*: 5'-GAAGCAGGGAGCTTTGAGG-3'	63°C	32	Hinf I
C677T	*Reverse*: 5'-ACGATGGGGCAAGTGATG-3'			
*ENOS*	*Forward*: 5'-CTGGAGATGAAGGCAGGAGAC-3'	56°C	35	Ban II
G894T	*Reverse*: 5'-CTCCATCCCACCCAGTCAATC-3'			
*ACE*	*Forward*: 5'-CTGGAGACCACTCCCATCCTTTCT-3'	55°C*	32	-
I/D	*Reverse*: 5'-GTCTCGATCTCCTGACCTCGTG-3'			
*AT1R*	*Forward*: 5'-AATGCTTGTAGCCAAAGTCACCT-3'	59°C	35	Dde I
A1166C	*Reverse*: 5'-GGCTTTGCTTTGTCTTGTTG-3'			
*LPL*	*Forward*: 5'-AAAATCAAGCAACCCTCAAG-3'	57°C*	35	Taq I
D9N	*Reverse*: 5'-TAGGGCAAATTTACTTGCGA-3'			
*APOC3*	*Forward*: 5'GGTGACCGATGGCTTCAGTT-3'	58°C	30	Sst I
SST I	*Reverse*: 5'-CAGAAGTGGATAGAGCGCT-3'			

* the touchdown PCR protocol was used for the ACE and LPL genes

### Statistical analysis

Sample size was estimated for comparisons between proportions, considering the polymorphism dominant model, an a error of 5%, a b power of 80%, a 95% confidence interval, and an expected proportion of 50%. The estimated sample size was of 90 participants. All analyses were performed using the SPSS 22.0 for Mac. Categorical variables were expressed as n (%) and compared between the genotypes using the Pearson’s chi-square test or the Fisher’s exact test, as appropriate. The chi-square test was used to analyze whether the distribution of observed and expected genotypic frequencies were in the Hardy-Weinberg equilibrium. Numerical variables were expressed as mean ± standard deviation or as median and interquartile range. The Kolmogorov - Smirnov test was used to evaluate whether the variables were normally distributed. For descriptive statistics, in case the variables were not normally distributed, the Student’s t test and the Mann Whitney test were used for unrelated samples. Pearson correlation was used to evaluate the correlations between the genetic score and the Gensini score. P-values lower than 0.05 were considered statistically significant.

## Results

A total of 116 patients were evaluated. Baseline characteristics of participants are described in Table 2.

**Table 2:** Demographic and clinical characteristics of participants

Variable	n = 116
Male (%)	74 (68)
Age (years)	56 ± 9
Hypertension (%)	104 (90)
Smoking (%)	35 (30)
Diabetes mellitus (%)	36 (31)
AMI (%)	55 (47)
Unstable angina (%)	61 (53)
Stroke (%)	9 (8)
PVD (%)	12 (10)
BMI (Kg/m^2^)	30.2 ± 4.7
Waist circumference (cm)	104.4 ± 10.8
Systolic arterial pressure (mmHg)	132 ± 24
Diastolic arterial pressure (mmHg)	86 ± 16
Heart rate (bpm)	68 ± 13
Gensini score (au)	21 (0-36)
Gensini ≥ median (%)	42 (51)
FMD (%)	13.7 ± 8.6
EIR (%)	14.9 (7.3-18.8)

Categorical variables expressed as N (%); numerical variables expressed as mean ± standard deviation or median and interquartile ranges. PVD: peripheral vascular disease; FMD: flow-mediated dilation; AMI: acute myocardial infarction; BMI: body mass index, EIR: endothelium‑independent relaxation; AU: arbitrary units.

Table 3 presents the distribution of allele and genotypic frequencies of the studied polymorphisms. The *PON-1, MTHFR* and *ENOS* genes were not in the Hardy-Weinberg equilibrium in the study population.

**Table 3:** Distribution of allele and genotypic frequencies for the polimorphisms of PON-1, MTHFR, ENOS, ECA, AT1R, APOC3 and LPL genes

Gene	Allele frequency		Genotypic frequency (%)			p*-*value (Hardy Weinberg)
PON-1	Q	R	QQ	QR	RR	
	0.63	0.37	36 (31)	76 (65)	4 (4)	0.0004
MTHFR	C	T	CC	CT	TT	
	0.61	0.39	35 (30)	72 (62)	9 (8)	0.0131
ENOS	G	T	GG	GT	TT	
	0.43	0.57	10 (9)	80 (69)	26 (22)	0.0006
ACE	I	D	II	ID	DD	
	0.33	0.67	17 (15)	42 (36)	57 (49)	0.1955
AT1R	A	C	AA	AC	CC	
	0.75	0.25	70 (60)	35 (30)	11 (10)	0.2351
APOC3	S1	S2	S1S1	S1S2	S2S2	
	0.16	0.84	2 (2)	33 (28)	81 (70)	0.8310
LPL	D	N	DD	DN	NN	
	0.34	0.66	17 (15)	45 (39)	54 (46)	0.3095

PON-1: paraoxonase-1; MTHFR: methylenotetrahydrofolate reductase; ENOS: endothelial nitric oxide synthase; ACE: angiotensin-converting enzyme; AT1R: angiotensin II type 1 receptor; APOC3: apolipoprotein C3; LPL: lipoprotein lipase. p < 0.05, chi-square test. Expected vs. observed genotypic frequencies were not in the Hardy‑Weinberg equilibrium in the studied population

### Distribution of clinical, demographic and laboratory characteristics

Results were analyzed by genetic polymorphism. The results are presented in details in electronic version in the Supplementary Tables 1-14 (access the link: http://www.arquivosonline.com.br/2018/english/11001/pdf/Tabelas_suplementares_Eng.pdf). For didactic purposes, data are presented by genotype, considering the R allele of *PON-1*, the T allele of *MTHFR*, the T allele of *ENOS*, the D allele of *ACE*, the C allele of *AT1R*, the S1 allele of *APOC3* and the N allele of *LPL* as risk alleles.

### Summary of findings

For the *PON-1* gene, hs-CRP (mg/dL) values were higher in the QQ genotype as compared with the QR/RR genotype [(12.8 (6.7-24.1) vs. 7.0 (4.8-16.5), p = 0.029] (Supplementary Tables 1 and 2), (access the link: http://www.arquivosonline.com.br/2018/english/11001/pdf/Tabelas_suplementares_Eng.pdf). For the *MTHFR* gene, HbA1-c (%) and adiponectin (ng/mL) levels were higher in the CT/TT genotype as compared with the CC genotype [6.0 (5.5-7.3) vs. 5.7 (5.2-6.6); p = 0.031 and 6.996 ± 5.032 vs. 4.990 ± 3.165; p = 0.015] (Supplementary Tables 3 and4), access the link: http://www.arquivosonline.com.br/2018/english/11001/pdf/Tabelas_suplementares_Eng.pdf. In addition, fasting glucose (mg/dL), HbA1c (%) and adiponectin levels (ng/mL) were higher in GT/TT vs. GG genotypes [(127 ± 48 vs. 106 ± 12, p = 0,001; 6,6 ± 1,9 vs. 5,9 ± 0,6, p = 0,028; 5010 (2688-10139) vs. 2148 (1912-3435), p = 0,011] (Supplementary Tables 5and6), (access the link: http://www.arquivosonline.com.br/2018/english/11001/pdf/Tabelas_suplementares_Eng.pdf). For the ACE gene, heart rate (bpm) was higher in the ID/DD genotype compared with the II genotype (71 ± 13 vs. 65 ± 11, p = 0.042 (Supplementary Tables 7 and 8), (access the link: http://www.arquivosonline.com.br/2018/english/11001/pdf/Tabelas_suplementares_Eng.pdf). Polymorphisms in the *AT1R* gene had no effect on clinical or laboratory variables (Supplementary Tables 9 and 10), (access the link: http://www.arquivosonline.com.br/2018/english/11001/pdf/Tabelas_suplementares_Eng.pdf).

For the APOC3 gene, FMD (%) was higher among S2S2 patients than in S1S1/S1S2 patients (14.7 ± 9.6 vs. 11.5 ± 5.2, p = 0.026) (Supplementary Tables 11 and 12), (access the link: http://www.arquivosonline.com.br/2018/english/11001/pdf/Tabelas_suplementares_Eng.pdf), and for the LPL gene, diastolic arterial pressure (mmHg) was lower in the DD genotype as compared with DN/NN (79 ± 15 vs. 87 ± 16, p = 0.043). In addition, a more severe degree of coronary atherosclerosis, evaluated by the Gensini score > median (%) value was found in patients with DD genotype compared with DN/NN (77% vs. 46%, p = 0.039) (Supplementary Tables 13 and 14) (access the link: http://www.arquivosonline.com.br/2018/english/11001/pdf/Tabelas_suplementares_Eng.pdf).

### Associations of genotypes with CAD extension and severity

A dominant model was assumed, as well as the score from 1 to 3 for the isolated genotypes - 1 for absence of risk allele; 2 for the presence of one risk allele; and 3 for the presence of 2 risk alleles in the same gene. Then, score 1 was assigned to the genotypes QQ of *PON-1*, CC of *MTHFR*, GG of *ENOS*, II of *ACE*, AA of *AT1R*, S2S2 of *APOC3* and DD of *LPL*. The score 2 was assigned to the genotypes QR of *PON-1*, CT of *MTHFR*, GT of *ENOS*, ID of *ACE*, AC of *AT1R*, S1S2 of *APOC3* and DN of *LPL*. The score 3 was assigned to the genotypes RR of *PON-1*, TT of *MTHFR*, TT of *ENOS*, DD of *ACE*, CC of *AT1R*, S1S1 of *APOC3* and NN of *LPL*.

The sum of the values assigned to each gene (7-21) yielded a genetic score, which was evaluated in absolute values and also in relation with the median (above or below) value. Correlations between genetic and Gensini scores were performed in absolute values and in relation to the median. Results are described in Table 4 and Supplementary Table 15 (access the link: http://www.arquivosonline.com.br/2018/english/11001/pdf/Tabelas_suplementares_Eng.pdf). Both genetic and Gensini scores had a normal distribution.

**Table 4:** Distribution of genetic score considering all polymorphisms of the PON-1, MTHFR, ENOS, ACE, AT1R), APOC3 and LPL genes

Genetic score	
N	116
Mean	13.3
Median	13.5
Standard deviation	1.58
Minimum	10
Maximum	17
Asymmetry	-0.263
Kurtosis	-0.146
P25	12.0
P75	14.0

PON-1: paraoxonase-1; MTHFR: methylenotetrahydrofolate reductase; ENOS: endothelial nitric oxide synthase; ACE: angiotensin-converting enzyme; AT1R: angiotensin II type 1 receptor; APOC3: apolipoprotein C3; LPL: lipoprotein lipase.

Genetic score tended to be higher in patients with a Gensini score < 50^th^ percentile (13.7 ± 1.5 vs. 13.0 ± 1.6; p = 0.066, Student’s t test for independent samples).

Gensini score was not different between genetic scores above and below the median (p50) [26 (0.00-44.00) vs. 18 (0.25-33.70), P=0.329]. In this population of patients with recent ACS and MS, a weak, inverse correlation was observed between the genetic and the Gensini scores (R = -0.194, p = 0.078, Pearson correlation coefficient).

## Discussion

The present study demonstrated that the genetic polymorphisms analyzed in patients with MS and recent ACS had a modest association with the severity of obstructive coronary disease. Only the DD genotype of D9N polymorphism of LPL was associated with higher prevalence of more severe coronary lesions. Analysis of the genetic score revealed that the combinations of the studied polymorphisms showed a trend of negative correlation with the anatomical extension of the coronary disease. This finding suggests that in these MS patients, coronary disease may be primarily associated with other mechanisms, with a strong environmental influence.

Many of the studied polymorphisms were associated with clinical and laboratory variables, such as heart frequency, diastolic arterial pressure, CRP, HbA1c and adiponectin.

A study involving six polymorphisms of LPL, including the D9N, showed that the severity of obstructive lesions, analyzed by the Gensini score, was associated with LPL haplotypes.^32^ Corsetti et al.^33^ showed an interaction of D9N polymorphism with Taq1B of CETP, which was a predictor of cardiovascular disease risk in women. In addition, *LPL* polymorphisms have been associated with increased concentrations of triglycerides;^34^ in our study, a trend towards higher values was found for the DD genotype as compared with the DN/NN genotype (p = 0.07), possibly due to the high prevalence of overweight/obesity and changes in glucose metabolism in these MS patients.

APOC3 polymorphism has an important role in the metabolism of triglyceride-rich lipoproteins and an influence on the development of CAD, particularly in MS and diabetes, with an association of haplotypes in the AI-C3-AIV gene cluster with coronary disease.^35^

Renin-angiotensin system genetic polymorphisms (I/D of *ACE* and *AT1R*) were not associated with CAD severity in our study. However, the ACE gene was previously associated with higher ACE levels in D/D patients with CAD,^36^ which was not confirmed in a larger sample.

In our study, the 192R allele of *PON-1* was associated with higher CRP levels, which is a biomarker of cardiovascular outcomes. In a large, three-year follow-up study (n = 3668), arylesterase activity, but not paraoxonase levels or PON-1 polymorphism, was associated with cardiovascular outcomes.^37^

For the *MTHFR* C677T polymorphism, a meta-analysis (n = 6912) demonstrated its association with early CAD,^38^ with higher homocysteine levels in the presence of T allele.^39^ In our study, CT/TT individuals had higher HbA1c and adiponectin levels as compared with CC individuals.

*ENOS* polymorphisms are involved in endothelial dysfunction. The T allele of the G894T polymorphism showed lower levels of nitric oxide and an association with CAD.^40^ The variant allele -786T (promoter region) was associated with CAD in a meta-analysis.^41^

Genetic score was inversely associated with greater extension and higher severity of CAD. In this population with MS, environmental factors may have an impact on the modulation of the expression of genes related to lipid metabolism, lipoprotein oxidation, endothelial function and blood pressure, and hence on atherogenesis.

## Conclusion

The studied polymorphisms had a small contribution to the extension of CAD. Only *LPL* D9N polymorphism was associated with CAD extension in patients with MS and recent ACS. Analysis of combined genetic polymorphisms showed a weak association with CAD extension, and an inverse relationship of genetic score with CAD extension and severity.

### Study limitations

The small sample size and the cross-sectional design may be considered limitations of our study. However, the exploratory aim of the study was to identify genetic polymorphisms that may be used in the identification of more severe atherosclerotic disease in this population of patients with MS and recent ACS. Its results contribute to the selection of genetic polymorphisms to be tested in prospective studies involving larger samples.
